# Ionic Liquid-Cured Epoxy/PCL Blends with Improved Toughness and Adhesive Properties

**DOI:** 10.3390/polym14132679

**Published:** 2022-06-30

**Authors:** Lidia Orduna, Iker Razquin, Itziar Otaegi, Nora Aranburu, Gonzalo Guerrica-Echevarría

**Affiliations:** POLYMAT and Department of Advanced Polymers and Materials, Physics, Chemistry and Technology, Faculty of Chemistry, University of the Basque Country (UPV/EHU), Paseo Manuel de Lardizábal 3, 20018 Donostia-San Sebastián, Spain; lidia.orduna@ehu.eus (L.O.); iker.razquin@ehu.eus (I.R.); itziar.otaegi@ehu.eus (I.O.); nora.aramburu@ehu.eus (N.A.)

**Keywords:** epoxy, ionic liquids, poly(ε-caprolactone), blends, adhesives, lap-shear, mechanical properties, toughness

## Abstract

In this work, ionic liquid (IL)-cured epoxy resins were modified by adding poly(ε-caprolactone) (PCL). Three different ILs were used in order to study how (a) the chemical structure of the ILs and (b) the PCL content affect the phase behaviour, microstructure, mechanical and adhesive properties. Regardless of the IL used or the PCL content, the obtained materials showed a single phase. The addition of PCL to the epoxy resin resulted in plasticizing of the network blends, lower glass transition temperatures (T_g_), and crosslinking densities (ν_e_). Low PCL contents did not have a significant impact on the mechanical properties. However, the adhesive properties improved significantly at low PCL contents. Higher PCL contents led to a significant increase in toughness, especially in the case of the imidazolium-based IL. The balance achieved between the mechanical and adhesive properties of these IL-cured epoxy/PCL blends constitutes an important step towards sustainability. This is because a biodegradable polymer (PCL) was used to substitute part of the epoxy resin, and the ILs—which are non-volatile and cure effectively at much lower contents—were used instead of conventional curing agents. Given the wide use of this kind of materials in the adhesive industry, the practical significance of these results must be emphasised.

## 1. Introduction

Epoxy resins are extensively used in a wide range of applications [1], including adhesives [2,3], coatings [4,5], and high-performance composites [6,7], among others. However, as one of the main drawbacks is their inherent brittleness, much research has gone into finding ways to increase their toughness. One of the most widely studied means of doing this has been by blending them with rubbers. However, blending with rubbers generally leads to a decrease in the modulus and strength [8], and the products from such blending are not suitable for high-temperature applications [9]. In addition, they usually need to be functionalized in order to bond them chemically with the epoxy resin [10]. The use of epoxidized oils has also been studied, notably soybean [11,12,13] and castor [14,15] oils. One of the easiest ways to improve the toughness of epoxy resins, however, is by adding thermoplastic polymers. Different factors such as the curing conditions [16,17,18], the curing agent [17,19,20,21,22], the amount [22,23] and the molecular weight of the thermoplastic polymer [20,21] have been seen to determine the miscibility of the components and the morphology of the resulting blends.

One of the first thermoplastic polymers blended with epoxy resins was polyether sulphone (PES) [20,21,22,24]. The use of different curing agents and PES with different molecular weights resulted in varying morphologies. Many other thermoplastic polymers have been used as epoxy modifiers and reported in the literature, including polyetherimide (PEI) [25], polymethylmethacrylate [26], polyethylene oxide [27], polycarbonate [28], and poly(ε-caprolactone) (PCL) [17,19,29,30,31,32,33,34,35,36,37,38].

Regarding the use of PCL, different morphologies have been reported depending on the curing agent employed. When anhydrides were used, the systems usually present phase separation above a critical molecular weight of PCL [30,31]. By contrast, the use of amines usually results in miscible blends [19,30,34,35,36,37]. This difference is due to the fact that amines can interact via hydrogen bonding with the PCL’s ester groups [23,30], which favours miscibility. Zheng et al. [38] reported miscible epoxy/PCL blends on the nanoscale, with homogeneity at the molecular level depending on the composition. However, depending on the curing conditions, thermoplastic concentration, and molecular weight [36], phase-separated morphologies have also been reported with PCL, such as in 4,4′-diaminodiphenylsulfone (DDS)-cured PCL/epoxy blends. Regarding mechanical properties, when used as a modifier, PCL successfully improved the toughness of the epoxy resin [19,32].

Furthermore, when added to epoxy resins, thermoplastics were also reported to improve the adhesive properties [39,40,41,42]. Kishi et al. [39] studied the T-peel adhesive strength of an epoxy resin with polyamide 12 pre-formed particles and reported a value three times higher than the neat resin. They attributed this to a crack bridging mechanism by the particles behind the crack-tip. Karthikeyan et al. [40] recently reported that the addition of 10% polyetheretherketone led to a 19% improvement in the lap shear strength. However, higher concentrations of thermoplastics resulted in a decrease in adhesion due to poor wettability. Ekrem et al. [41] studied the adhesive strength of an epoxy resin modified with an electrospun nanofiber mat of polyvinyl alcohol (PVA), and reported a 13.5% improvement in the shear strength. For an epoxy resin modified with a hyperbranched polymer, Buonocore et al. [42] obtained a 27.6% improvement in the lap shear strength. They attributed this result to enhanced interaction between the end group of the hyperbranched polymer and the substrate.

Regarding the adhesive properties of epoxy/PCL blends, Arnebold et al. [43] obtained moderate increases (≈ 50%) in the tensile shear strength by adding either a reactive or a non-reactive PCL to the epoxy resin. Luo et al. [44,45] used immiscible PCL as the adhesive component in a previously cured phase-separated epoxy/PCL blend.

Furthermore, in the search for sustainable materials, ionic liquids are being studied as substitutes for traditional toxic and volatile epoxy curing agents. Imidazolium [46,47,48,49,50,51,52,53] and phosphonium-based [46,54,55,56,57,58] ionic liquids have been reported, among others, to cure the epoxy resin effectively. These agents normally initiate the curing reaction by opening the epoxy rings, thus leading to homopolymerization. Different mechanisms have been reported in the literature, as can be seen in Table 1.

In the literature, few studies have focused on the use of ionic liquids as curing agents for epoxy/thermoplastic blends [60,61]. Nguyen et al. [60] compared the flexural properties and toughness of a DGEBA/poly(2,6-dimethyl-1,4-phenylen ether) (PPE) system cured with an amine and two ionic liquids, trihexyltetradecylphosphonium bis(2,4,4-trimethylpentyl)phosphinate (IL-P-TMPP) and tributhylethyl phosphonium diethylphosphate (IL-P-DEP). Halawani et al. [61] compared the toughness of DDS-cured and IL-P-DEP-cured DGEBA/PEI blends. In this work, we describe the use of ionic liquids as curing agents in epoxy/PCL blends. Based on a previous work [62], three ionic liquids were used to study how the chemical structure of the IL and the PCL concentration impact the phase behaviour, microstructure, mechanical and adhesive properties of these materials.

## 2. Materials and Methods

### 2.1. Materials

Diglycidyl ether of bisphenol A (DGEBA) (Nazza, Eurotex) with an epoxy equivalent of 186 g/equivalent was the epoxy resin used in this study. Poly(ε-caprolactone) (PCL) commercially known as CAPA6800 (80,000 g/mol) (Ingevity) was supplied by Ravago Chemicals. 1-Ethyl-3-methylimidazolium dicyanamide (IL-I-DCA) and trihexyltetradecylphosphonium bis(2,4,4-trimethylpentyl)phosphinate (IL-P-TMPP) were acquired from Sigma Aldrich. Trihexyltetradecylphosphonium dicyanamide (IL-P-DCA) was purchased from IoLiTec-Ionic Liquid Technologies GmbH. Table 2 shows the chemical structure of the materials.

### 2.2. Preparation of Samples

**Figure 1:**
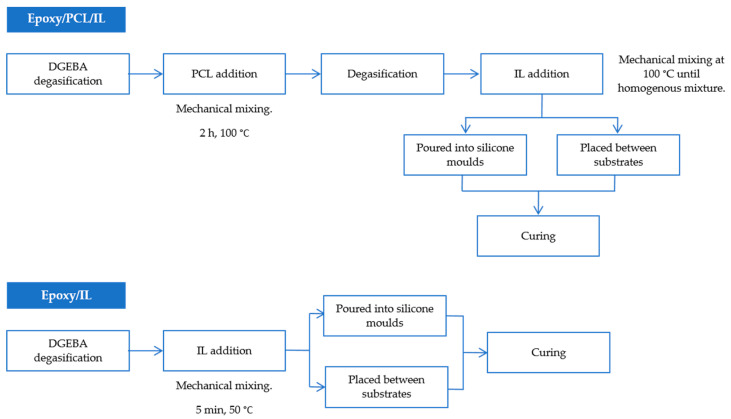
Experimental flowchart for the preparation of the samples.

Prior to the preparation of the samples, the DGEBA was degassed in a vacuum oven at 80 °C for 1 h. Next, the epoxy resin and the PCL (10, 20 and 30 wt% concentrations) were mechanically mixed at 100 °C for two hours. After degassing the corresponding mixture in a vacuum oven, it was heated to 100 °C and 10 parts per hundred resin (phr) of ionic liquid was added. The mixture was mechanically stirred until it became homogenous. Finally, in order to produce the test specimens, it was poured into silicone moulds, and the corresponding curing protocol for each ionic liquid was followed (Table 3). To prepare the samples for the lap shear tests, the mixture was placed between substrates covering an area of 12.5 mm × 25 mm and the samples were cured. DGEBA/IL samples were also obtained as a reference. For these samples, the two components were mechanically mixed at 50 °C for 5 min. The mixture was then either poured into silicone moulds in order to obtain test specimens or placed between substrates for the lap shear tests and the appropriate curing protocol was followed. All this information is shown schematically in Figure 1.

### 2.3. Characterization

#### 2.3.1. Phase Behaviour

Differential scanning calorimetry (DSC) was conducted using a Perkin-Elmer DSC-7 calorimeter calibrated using an indium standard as a reference. Samples were taken from previously cured specimens and were heated from 30 °C to 250 °C at a rate of 20 °C/min under a nitrogen atmosphere. 

Dynamic mechanical analysis (DMA) was performed on rectangular specimens in a TA Q800 viscoelastometer in single cantilever bending mode. The temperature interval was set from −100 °C to 250 °C, and the heating rate at 4 °C/min. The tests were carried out at a frequency of 1 Hz.

In order to calculate the crosslinking density of the samples, the elasticity theory was used (Equation (1)) [49,55].

(1) $$\nu_{e}=\frac{E_{r}}{3RT_{r}}$$

where $E_{r}$ is the storage modulus in the rubbery state, *T_r_* is the temperature corresponding to the $E_{r}$ value (*T* = 245 °C) and R is the ideal gas constant (*R* = 8.314 J/mol K).

#### 2.3.2. Microstructure

The morphology of the samples was analysed with a Hitachi TM3030Plus scanning electronic microscope (SEM) using an accelerating voltage of 15 kV and a secondary electron detector. Two-mm-thick rectangular specimens were cryogenically broken by bending after 2 h in liquid nitrogen and coated with gold before being observed.

#### 2.3.3. Mechanical Properties

The mechanical properties of the IL-cured epoxy/PCL blends were determined by bending tests. An Instron 5569 universal testing machine equipped with a three-point bending device was used. The crosshead speed was set at 2 mm/min and the span at 64 mm. The samples measured 80 mm × 10 mm × 4 mm, in compliance with the ISO 178 standard. A minimum of 5 specimens were tested for each composition.

The flexural strength (*σ_F_*) and the deformation at break (*ε_F_*) were calculated using Equations (2) and (3), respectively, according to ISO 178. The flexural modulus (*E_f_*) was calculated from the slope of the tangent line of the linear zone within the elastic limit of the stress-strain curve (Equation (4)).

(2) $$\sigma_{F}=\frac{3F_{max}L}{2bh^{2}}$$

(3) $$\varepsilon_{F}\;\left(\%\right)=\frac{6sh}{L^{2}}\times 100$$

(4) $$E_{f}=\frac{FL^{3}}{4sbh^{3}}$$

where $F_{max}$ and F are the maximun load and the load, respectively, L is the span, b is the width of the specimen, h the thickness and s the deflection.

Charpy impact tests were used to evaluate the impact strength of the samples. The tests were carried out in a Ceast 6548/00 impact tester using a 2 J pendulum. Notched specimens (depth 2.54 mm and radius 0.25 mm) were used. A minimum of eight impact specimens were tested for each reported value.

#### 2.3.4. Adhesive Properties

Lap shear tests were carried out to study the adhesive properties of the obtained materials. Aluminium 2021-T351 alloy sheets measuring 100 mm × 25 mm × 1.6 mm, purchased from Rocholl GmbH, were used as substrates. The geometry and adhesion area were set according to ASTM D-1002 (12.5 mm × 25 mm) and the substrates were cleaned with acetone before use. 

An Instron 5569 with a rate of 1 mm/min was used to carry out the tests. The lap shear strength was calculated by dividing the maximum force (*F_max_*) by the adhesion area. 10 specimens were tested for each reported value.

## 3. Results and Discussion

### 3.1. Phase Behaviour 

**Figure 2:**
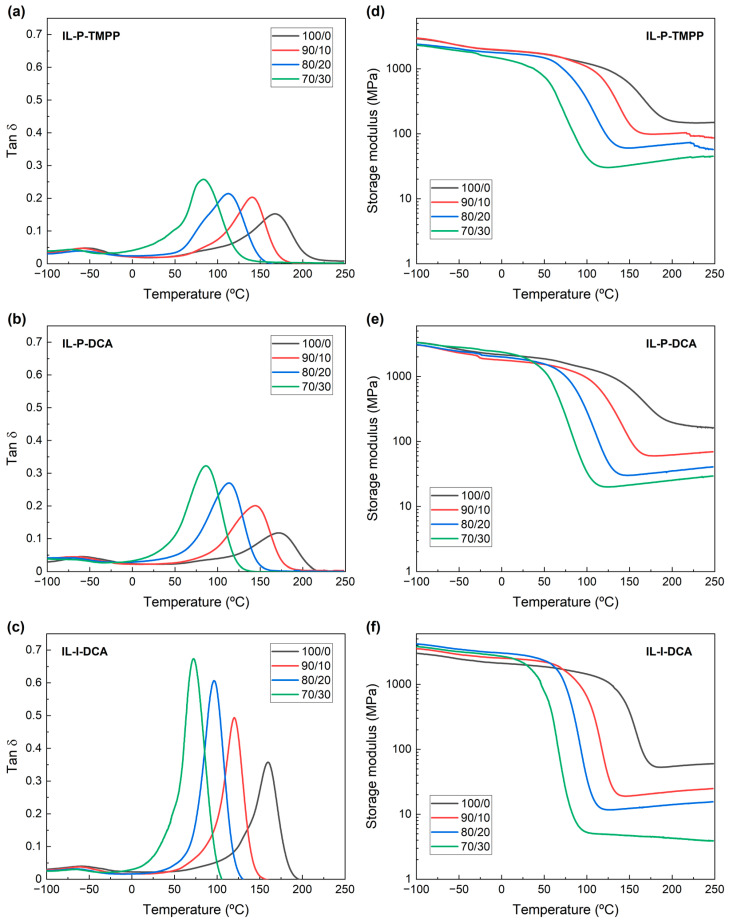
Tan δ (**a**) IL-P-TMPP, (**b**) IL-P-DCA and (**c**) IL-I-DCA) and storage modulus (**d**) IL-P-TMPP, (**e**) IL-P-DCA and (**f**) IL-I-DCA) vs. T curves obtained by DMA for the DGEBA/PCL blends at different PCL contents.

Figure 2 shows the tan δ and storage modulus vs. temperature curves of the samples cured with the different ionic liquids, at different PCL contents. As can be seen in Figure 2a–c, regardless of either the IL used or the PCL content, the tan δ curves showed one single main peak, corresponding to the α transition, which is related to the T_g_. The T_g_ and crosslinking density values extracted from these curves are summarized in Table 4. As can also be seen in Figure 2a–c and Table 4, the position of this peak decreased with increasing PCL contents. The peak at low temperatures (≈−50 °C) corresponds to the β transition of the DGEBA, which is associated with the movement of the -CH_2_-CHOH-CH_2_-O-segment [63,64].

The appearance of a single tan δ peak points to the epoxy/PCL blend being single-phase, probably due to the miscibility of DGEBA and PCL in the studied compositions [17,19,30]. Transesterification reactions may also be considered as they have also been reported for epoxy/PCL systems, mainly for cationically-cured ones [43,65,66] where the PCL became chemically anchored in the network [35]. Either way, a decrease in the T_g_ and the crosslinking density (Table 4) resulting from the addition of PCL has also been observed in epoxy/PCL systems cured with conventional curing agents [17,19,30,35], and has been attributed to the plasticizing effect of the PCL chains in the epoxy resin network. Regarding the impact of the type of ionic liquid on the phase behaviour of the epoxy/PCL blend, Figure 2 and Table 4 show very similar trends in all three as the PCL content increased. This is consistent with the widely reported role of ILs in the curing reaction of epoxy resins, as they open the epoxy ring, initiating the homopolymerization of the resin without modifying the chemical structure of the network.

**Figure 3:**
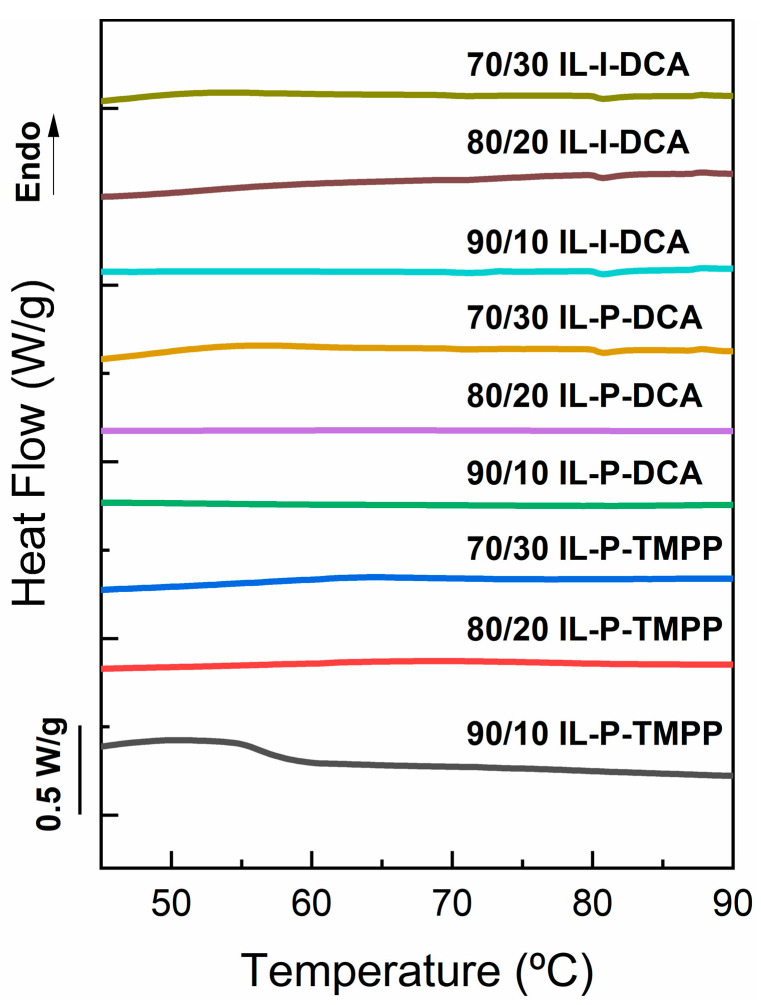
DSC scans of the IL-cured epoxy/PCL blends. The curves have been shifted in the *Y*-axis for clarity.

DSC analysis was carried out to verify whether crystallization of the PCL had taken place in the cured epoxy/PCL blends. Figure 3 shows the heat flow vs. temperature curves for the different ILs and PCL contents. As can be seen, no melting peak appeared in any of the samples studied, which is indicative of the PCL not crystallizing in the DGEBA/PCL blends.

The literature shows that in miscible epoxy/thermoplastic blends, the thermoplastic only crystallizes when its domains have reached a minimum required size. Slight crystallizations have also been observed as a consequence of minor phase separation [19]. Moreover, in both miscible [34] and transesterified [67] epoxy/PCL systems, it has been proposed that the high T_g_ of the samples (higher than the crystallization temperature of the PCL) restricts the movement of the PCL chains which is necessary for crystallization to take place. Therefore, the lack of PCL crystallization provides additional evidence of the presence of a single phase in the blends in this study, regardless of the IL or the PCL content used.

### 3.2. Microstructure 

**Figure 4:**
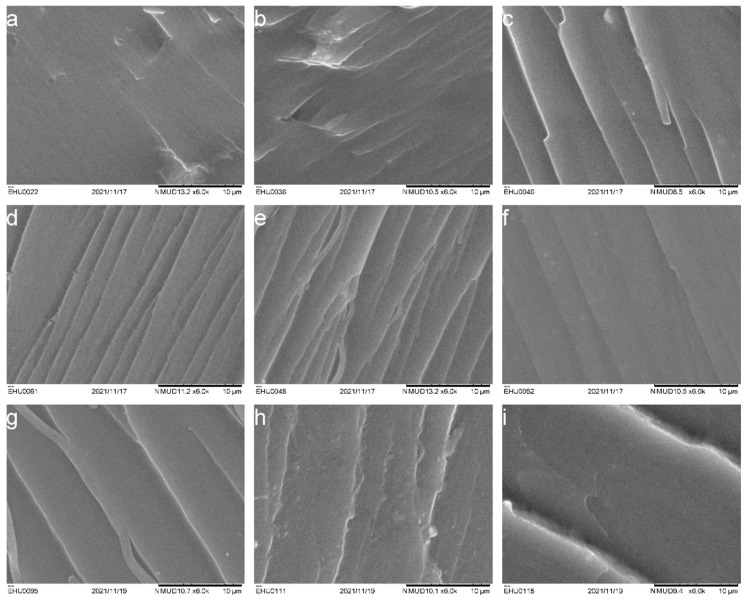
SEM micrographs of cryogenically broken specimens with different PCL contents: (**a**) 90/10 IL-P-TMPP, (**b**) 80/20 IL-P-TMPP, (**c**) 70/30 IL-P-TMPP, (**d**) 90/10 IL-P-DCA, (**e**) 80/20 IL-P-DCA, (**f**) 70/30 IL-P-DCA, (**g**) 90/10 IL-I-DCA, (**h**) 80/20 IL-I-DCA, and (**i**) 70/30 IL-I-DCA.

Figure 4 shows SEM micrographs of the cryogenically broken specimens of the IL-cured epoxy/PCL blends at different PCL contents. As can be observed, all the studied blends are homogenous and single-phase, which is fully consistent with the DMA and DSC results.

Uncured epoxy/PCL blends are known to be fully miscible in the amorphous state, i.e., above the melting temperature of PCL, regardless of the PCL content [37,68], which indicates that if phase-separated cured blends are obtained, phase separation takes place as a consequence of curing the epoxy resin. Both single-phase [19,30,34,35,36,37] and phase-separated [16,19,23,31,32] epoxy/PCL blends have been reported in the literature when conventional curing agents were used. In fact, miscible [34,35] and immiscible [61,69] blends have also been reported even when the same curing agent was used. This is because phase separation depends on both thermodynamics and kinetics, and therefore not only on the curing agent but also on the curing conditions, the PCL concentration, and even its molecular weight.

When considering IL-cured epoxy/thermoplastic blends, Halawani et al. [61] also observed that epoxy/PEI blends cured with IL-P-DEP showed a single-phase morphology, which they related to the IL being able to dissolve the thermoplastic polymer.

### 3.3. Mechanical Properties 

**Figure 5:**
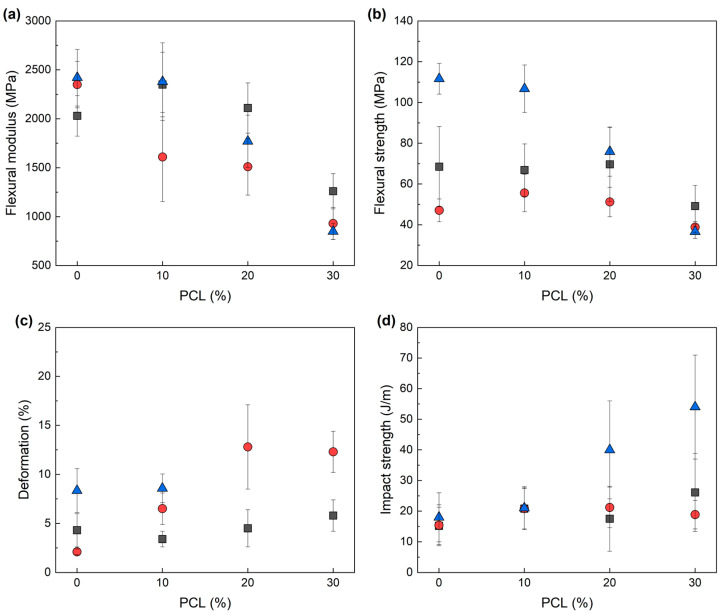
Flexural modulus (**a**), flexural strength (**b**), deformation at break (**c**), and impact strength (**d**) of IL-P-TMPP-(■), IL-P-DCA-(●), and IL-I-DCA-(▲) cured epoxy/PCL blends as a function of the PCL content. Deformation at break values of the IL-I-DCA-cured blends containing 20% and 30% of PCL are not shown, as the samples did not break.

The effect of the addition of PCL on the mechanical properties of the IL-cured epoxy/PCL blends was studied by bending and impact tests. The results are shown in Figure 5 and summarized in Table 5. As can be observed, both the chemical structure of the IL used to cure the samples and the amount of added PCL affected the mechanical properties.

With respect to low-strain mechanical properties, Figure 5 and Table 5 show how increasing amounts of added PCL led to a gradual decrease in the flexural modulus and strength. This was to be expected given the mechanical properties of neat PCL and the findings in the literature [19,31,32]. These decreases varied depending on the IL used, with IL-I-DCA producing the greatest drop. For this system and high PCL contents (30%), the flexural modulus and strength appeared at even lower values than those predicted by the rule of mixtures, which is consistent with the low T_g_ and crosslinking density values shown in Table 4. By contrast, at low PCL contents (10% and 20%), most of the compositions showed only moderate decreases and values close to those of the pure epoxy resin and well above the linear behaviour. However, the high standard deviations obtained for these measurements must also be considered, reducing the significance of the trends observed. Regarding high-strain mechanical properties, as expected, the addition of PCL to the epoxy resin led to a gradual increase in the ductility and impact strength, although the behaviour varied depending on the type of IL used to cure the epoxy resin. As can be seen in Figure 5c, the ductility increased at high PCL contents irrespective of the IL used, but this increase was barely significant in the case of IL-P-TMPP, considerable in the case of IL-P-DCA, and outstanding in the case of IL-I-DCA where the samples bent and slipped during the test, without breaking.

Figure 5d shows that the impact strength behaved in a similar way to the ductility: the impact strength of the blends with high PCL contents improved regardless of the IL used to cure the epoxy resin. As for the ductility, the IL-I-DCA-cured samples showed outstanding results. The 30% PCL IL-I-DCA-cured epoxy resin, for example, showed a 200% increase compared to the impact strength of the pure epoxy resin. Therefore, PCL has a similar toughening effect on these IL-cured epoxy resins to that of previously studied epoxy/PCL blends cured with conventional curing agents [17,32]. The chemical structure of the IL plays an important role in the toughening effect of the PCL and the overall mechanical properties of the epoxy/PCL blends, with the IL-I-DCA-cured material showing the best balance of properties.

For example, the IL-I-DCA-cured 80/20 epoxy/PCL composition in this study showed a 110% increase in impact strength, was ductile (did not break in the bending test), and had a 1% decrease in flexural strength and a 20% decrease in modulus compared to the conventional amine-cured pure epoxy resin which was also prepared and tested in this work (DGEBA/Aradur 2954 system, 19 J/m impact strength, 4.8% elongation at break, 76.6 MPa flexural strength, and 2210 MPa flexural modulus).

### 3.4. Adhesive Properties 

**Figure 6:**
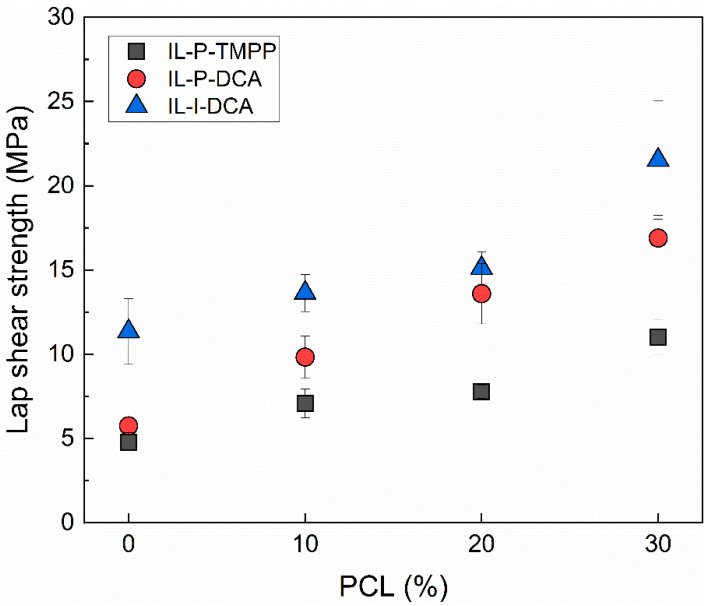
Lap shear strength of the IL-cured epoxy/PCL blends as a function of the PCL content.

The effect of adding PCL to IL-cured epoxy resins on the adhesive properties was studied using lap shear tests. Figure 6 shows the lap shear strength of all the compositions studied. As can be seen, irrespective of the IL in question, the addition of PCL led to an improvement in the adhesive properties of the epoxy resin, with higher lap shear strength at increasing PCL contents. The values of the epoxy/PCL 70/30 compositions were 129%, 196%, and 88% higher than that of the corresponding pure epoxy in the case of IL-P-TMPP, IL-P-DCA, and IL-I-DCA, respectively. An absolute value of 21.5 ± 3.5 MPa was obtained for the maximum PCL content and the imidazolium-based IL. When this value is compared to that of the epoxy resin cured with a conventional amine-based curing agent (DGEBA/Aradur 2954, 6.8 MPa), a 216% increase was observed. The improvement of the adhesive properties of conventionally-cured epoxy resins through the addition of thermoplastics, rubbers, and epoxidized oils has previously been reported in the literature and attributed to lower crosslinking densities and/or improved toughness [39,40,41,42,70,71,72,73,74,75,76,77,78,79], which is fully consistent with the results obtained in the present work (Table 4, Figure 5d).

Their high lap shear strength makes these IL-cured epoxy/PCL blends suitable for use as adhesives. Of particular additional interest is the fact that they are more sustainable than traditional epoxy-amine systems. 

## 4. Conclusions

Epoxy/PCL blends cured with three different ionic liquids were prepared with a view to studying the effect of the chemical structure of the ILs and the PCL concentration on their phase behaviour, microstructure, mechanical and adhesive properties. Characterization by DMA, DSC, and SEM corroborated the single-phase structure of the blends. The addition of PCL led to a decrease in the T_g_ and the crosslinking density of the samples due to its plasticizing effect on the network, which was also detected by bending and impact tests. The addition of low concentrations of PCL did not significantly affect the mechanical properties of the IL-cured epoxy resins while larger amounts led to increases in both the ductility and the toughness of the materials. This effect was more pronounced in the imidazolium-based IL-cured system. The adhesive properties were also notably improved by adding PCL, due to the decrease in the crosslinking density and the toughening effect. As a result, the materials obtained in this study represent a more sustainable alternative to traditional epoxy systems. This is not only because PCL—which is biodegradable—replaces some of the DGEBA, or because ionic liquids are non-volatile and a significantly smaller amount is needed to effectively cure the epoxy resin, but also because the mechanical-adhesive behaviour of these systems is well-balanced. On the one hand, while high strain mechanical properties, particularly toughness, significantly improved, the effect on the low strain ones was limited. On the other hand, the adhesion values increased up to 200% with respect to the reference ones. Given the wide use of this kind of materials in the adhesives industry, the practical implications of these results are noteworthy.

## Figures and Tables

**Table 1:** Proposed initiation mechanisms for curing epoxy resins using ILs.

IL Type	Initiation Mechanism	Reference
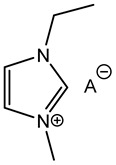	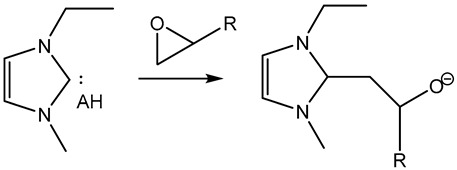	[59]
	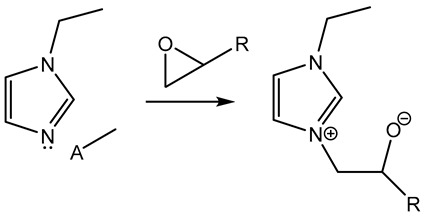	[49,59]
	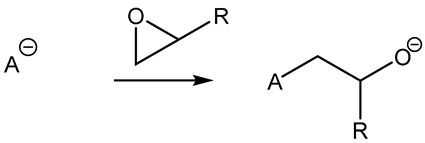	[59]
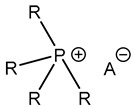	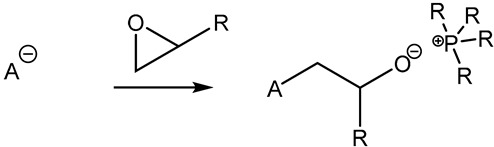	[56,57,58]

**Table 2:** Chemical structure of the materials studied.

Material	Structure
DGEBA	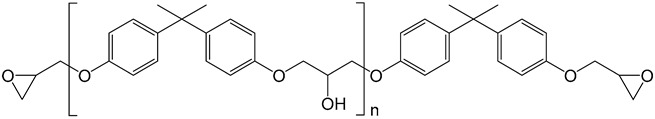
PCL	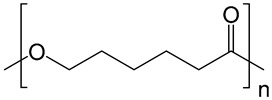
IL-P-TMPP	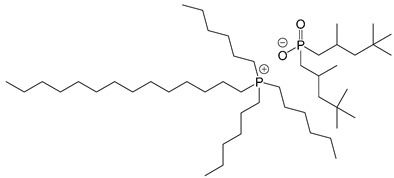
IL-P-DCA	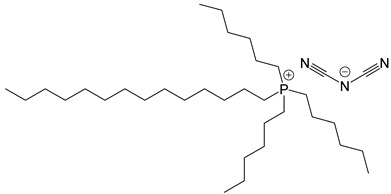
IL-I-DCA	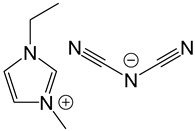

**Table 3:** Curing protocols for the ILs.

Curing Agent	Concentration	Curing Protocol
IL-P-TMPP	10 phr	2 h 80 °C/2 h 120 °C/1 h 150 °C/1 h 170 °C
IL-P-DCA	10 phr	2 h 120 °C/2 h 140 °C/1 h 170 °C
IL-I-DCA	10 phr	2 h 110 °C/1 h 140 °C/1 h 170 °C

**Table 4:** T_g_ and crosslinking densities obtained from the DMA analysis.

IL	PCL (%)	T_g_ (°C)	ν_e_ (mol/m^3^)
IL-P-TMPP	0	168	11,509
	10	141	6756
	20	112	4487
	30	83	3462
IL-P-DCA	0	172	12,616
	10	144	5338
	20	113	3117
	30	86	2252
IL-I-DCA	0	160	4625
	10	120	1912
	20	96	1197
	30	72	302

**Table 5:** Mechanical properties of the IL-cured epoxy/PCL blends.

IL	PCL (%)	Flexural Modulus (MPa)	Flexural Strength (MPa)	Deformation at Break (%)	Impact Strength (J/m)
IL-P-TMPP	0	2030 ± 210	68.5 ± 19.7	4.3 ± 1.7	15 ± 6
	10	2350 ± 330	66.8 ± 2.0	3.4 ± 0.8	21 ± 7
	20	2110 ± 260	69.7 ± 11.3	4.5 ± 1.9	18 ± 11
	30	1260 ± 180	49.2 ± 2.9	5.8 ± 1.6	26 ± 13
IL-P-DCA	0	2350 ± 240	47.1 ± 5.6	2.1 ± 0.3	15 ± 7
	10	1610 ± 450	55.6 ± 9.2	6.5 ± 1.6	21 ± 7
	20	1510 ± 290	51.2 ± 7.2	12.8 ± 4.4	21 ± 7
	30	930 ± 160	38.8 ± 2.8	12.3 ± 2.1	19 ± 5
IL-I-DCA	0	2420 ± 290	111.6 ± 7.6	8.3 ± 2.2	18 ± 8
	10	2380 ± 400	106.8 ± 11.7	8.6 ± 1.5	21 ± 7
	20	1770 ± 270	75.9 ± 12.1	*	40 ± 16
	30	850 ± 80	36.6 ± 3.3	*	54 ± 17

* Samples did not break.

## Data Availability

Not applicable.

## References

[1] Jin F.-L., Li X., Park S.-J. (2015). Synthesis and application of epoxy resins: A review. J. Ind. Eng. Chem..

[2] Ahmadi Z. (2019). Nanostructured epoxy adhesives: A review. Prog. Org. Coat..

[3] Maggiore S., Banea M.D., Stagnaro P., Luciano G. (2021). A review of structural adhesive joints in hybrid joining processes. Polymers.

[4] Verma C., Olasunkanmi L.O., Akpan E.D., Quraishi M.A., Dagdag O., El Gouri M., Sherif E.-S.M., Ebenso E.E. (2020). Epoxy resins as anticorrosive polymeric materials: A review. React. Funct. Polym..

[5] Kausar A. (2020). Performance of corrosion protective epoxy blend-based nanocomposite coatings: A review. Polym. Plast. Technol. Mater..

[6] Wazalwar R., Sahu M., Raichur A.M. (2021). Mechanical properties of aerospace epoxy composites reinforced with 2D nano-fillers: Current status and road to industrialization. Nanoscale Adv..

[7] Dong M., Zhang H., Tzounis L., Santagiuliana G., Bilotti E., Papageorgiou D.G. (2021). Multifunctional epoxy nanocomposites reinforced by two-dimensional materials: A review. Carbon.

[8] Jayan J.S., Saritha A., Joseph K. (2018). Innovative materials of this era for toughening the epoxy matrix: A review. Polym. Compos..

[9] Ratna D., Banthia A.K. (2000). Toughened epoxy adhesive modified with acrylate based liquid rubber. Polym. Int..

[10] Ratna D., Banthia A.K. (2004). Rubber toughened epoxy. Macromol. Res..

[11] Chen Y., Yang L.T., Wu J.H., Ma L.J., Finlow D.E., Lin S.Q., Song K.M. (2013). Thermal and mechanical properties of epoxy resin toughened with epoxidized soybean oil. J. Therm. Anal. Calorim..

[12] Ratna D. (2001). Mechanical properties and morphology of epoxidized soyabean-oil-modified epoxy resin. Polym. Int..

[13] Park S.J., Jin F.L., Lee J.R. (2004). Thermal and mechanical properties of tetrafunctional epoxy resin toughened with epoxidized soybean oil. Mater. Sci. Eng..

[14] Paluvai N.R., Mohanty S., Nayak S.K. (2015). Fabrication and evaluation of acrylated epoxidized castor oil-toughened diglycidyl ether of bisphenol A nanocomposites. Can. J. Chem. Eng..

[15] Park S.J., Jin F.L., Lee J.R. (2004). Effect of biodegradable epoxidized castor oil on physicochemical and mechanical properties of epoxy resins. Macromol. Chem. Phys..

[16] Chen J.L., Chang F.C. (2001). Temperature-dependent phase behavior in poly(ε-caprolactone)-epoxy blends. Polymer.

[17] Luo X., Liu X.-F., Ding X.-M., Chen L., Chen S.-C., Wang Y.-Z. (2021). Effects of curing temperature on the structure and properties of epoxy resin-poly(ε-caprolactam) blends. Polymer.

[18] Remiro P.M., Marieta C., Riccardi C., Mondragon I. (2001). Influence of curing conditions on the morphologies of a PMMA-modified epoxy matrix. Polymer.

[19] Parameswaranpillai J., Sidhardhan S.K., Jose S., Hameed N., Salim N.V., Siengchin S., Pionteck J., Magueresse A., Grohens Y. (2016). Miscibility, phase morphology, thermomechanical, viscoelastic and surface properties of poly(ε-caprolactone) modified epoxy systems: Effect of curing agents. Ind. Eng. Chem. Res..

[20] Raghava R.S. (1988). Development and characterization of thermosetting thermoplastic polymer blends for applications in damage-tolerant composites. J. Polym. Sci. B Polym. Phys..

[21] Raghava R.S. (1987). Role of matrix-particle interface adhesion on fracture-toughness of dual phase epoxy-polyethersulfone blend. J. Polym. Sci. B Polym. Phys..

[22] Bucknall C.B., Partridge I.K. (1986). Phase-separation in cross-linked resins containing polymeric modifiers. Polym. Eng. Sci..

[23] Chen J.L., Chang F.C. (1999). Phase separation process in poly(ε-caprolactone)-epoxy blends. Macromolecules.

[24] Bucknall C.B., Partridge I.K. (1983). Phase-separation in epoxy-resins containing polyethersulfone. Polymer.

[25] Bucknall C.B., Gilbert A.H. (1989). Toughening tetrafunctional epoxy-resins using polyetherimide. Polymer.

[26] Das B., Chakraborty D., Hajra A.K., Sinha S. (1994). Epoxy poly(methyl methacrylate) interpenetrating polymer networks morphology, mechanical and thermal-properties. J. Appl. Polym. Sci..

[27] Guo Q.P., Peng X.S., Wang Z.J. (1991). The miscibility and morphology of epoxy resin poly(ethylene oxide) blends. Polymer.

[28] Chen M.C., Hourston D.J., Sun W.B. (1992). Miscibility and fracture-behavior of an epoxy-resin bisphenol-a polycarbonate blend. Eur. Polym. J..

[29] Barone L., Carciotto S., Cicala G., Recca A. (2006). Thermomechanical properties of epoxy/poly(ε-caprolactone) blends. Polym. Eng. Sci..

[30] Clark J.N., Daly J.H., Garton A. (1984). Hydrogen-bonding in epoxy-resin poly(ε-caprolactone) blends. J. Appl. Polym. Sci..

[31] Cohades A., Manfredi E., Plummer C.J.G., Michaud V. (2016). Thermal mending in immiscible poly(ε-caprolactone)/epoxy blends. Eur. Polym. J..

[32] Siddhamalli S.K. (2000). Toughening of epoxy/polycaprolactone composites via reaction induced phase separation. Polym. Compos..

[33] Noshay A., Robeson L.M. (1974). Epoxy/modifier block copolymers. J. Polym. Sci. A Polym. Chem..

[34] Guo Q., Harrats C., Groeninckx G., Reynaers H., Koch M.H.J. (2001). Miscibility, crystallization and real-time small-angle X-ray scattering investigation of the semicrystalline morphology in thermosetting polymer blends. Polymer.

[35] Chen J.L., Huang H.M., Li M.S., Chang F.C. (1999). Transesterification in homogeneous poly(ε-caprolactone)-epoxy blends. J. Appl. Polym. Sci..

[36] Ni Y., Zheng S.X. (2005). Influence of intramolecular specific interactions on phase behavior of epoxy resin and poly(ε-caprolactone) blends cured with aromatic amines. Polymer.

[37] Zheng S., Zheng H., Guo Q. (2003). Epoxy resin/poly(ε-caprolactone) blends cured with 2,2-bis[4-(4-aminophenoxy)phenyl]propane. I. Miscibility and crystallization kinetics. J. Polym. Sci. B Polym. Phys..

[38] Zheng S., Guo Q., Chan C.-M. (2003). Epoxy resin/poly(ε-caprolactone) blends cured with 2,2-bis[4-(4-aminophenoxy)phenyl]propane. II. Studies by Fourier transform infrared and carbon-13 cross-polarization/magic-angle spinning nuclear magnetic resonance spectroscopy. J. Polym. Sci. B Polym. Phys..

[39] Kishi H., Uesawa K., Matsuda S., Murakami A. (2005). Adhesive strength and mechanisms of epoxy resins toughened with pre-formed thermoplastic polymer particles. J. Adhes. Sci. Technol..

[40] Karthikeyan L., Robert T.M., Mathew D., Suma D.D., Thomas D. (2021). Novel epoxy resin adhesives toughened by functionalized poly (ether ether ketone) s. Int. J. Adhes. Adhes..

[41] Ekrem M., Avci A. (2018). Effects of polyvinyl alcohol nanofiber mats on the adhesion strength and fracture toughness of epoxy adhesive joints. Compos. Part B Eng..

[42] Buonocore G.G., Schiavo L., Attianese I., Borriello A. (2013). Hyperbranched polymers as modifiers of epoxy adhesives. Compos. Part B Eng..

[43] Arnebold A., Wellmann S., Hartwig A. (2016). Partially crystalline epoxy networks with superior mechanical and adhesion properties. J. Adhes. Sci. Technol..

[44] Luo X.F., Ou R.Q., Eberly D.E., Singhal A., Viratyaporn W., Mather P.T. (2009). A thermoplastic/thermoset blend exhibiting thermal mending and reversible adhesion. ACS Appl. Mater. Interfaces.

[45] Luo X.F., Lauber K.E., Mather P.T. (2010). A thermally responsive, rigid, and reversible adhesive. Polymer.

[46] Maka H., Spychaj T., Zenker M. (2015). High performance epoxy composites cured with ionic liquids. J. Ind. Eng. Chem..

[47] Maka H., Spychaj T., Sikorski W. (2014). Deep eutectic ionic liquids as epoxy resin curing agents. Int. J. Polym. Anal. Charact..

[48] Maka H., Spychaj T. (2012). Epoxy resin crosslinked with conventional and deep eutectic ionic liquids. Polimery.

[49] Maka H., Spychaj T., Pilawka R. (2012). Epoxy resin/ionic liquid systems: The influence of imidazolium cation size and anion type on reactivity and thermomechanical properties. Ind. Eng. Chem. Res..

[50] Kowalczyk K., Spychaj T. (2003). Ionic liquids as convenient latent hardeners of epoxy resins. Polimery.

[51] Rahmathullah M.A.M., Jeyarajasingam A., Merritt B., VanLandingham M., McKnight S.H., Palmese G.R. (2009). Room temperature ionic liquids as thermally latent initiators for polymerization of epoxy resins. Macromolecules.

[52] Yin Y., Liu M.H., Wei W., Zheng C.M., Gao J., Zhang W.X., Zheng C.B., Deng P.Y., Xing Y. (2018). DGEBA/imidazolium ionic liquid systems: The influence of anions on the reactivity and properties of epoxy systems. J. Adhes. Sci. Technol..

[53] Maksym P., Tarnacka M., Dzienia A., Matuszek K., Chrobok A., Kaminski K., Paluch M. (2017). Enhanced polymerization rate and conductivity of ionic liquid-based epoxy resin. Macromolecules.

[54] Leclere M., Livi S., Marechal M., Picard L., Duchet-Rumeau J. (2016). The properties of new epoxy networks swollen with ionic liquids. RSC Adv..

[55] Nguyen T.K.L., Livi S., Soares B.G., Pruvost S., Duchet-Rumeau J., Gerard J.-F. (2016). Ionic liquids: A new route for the design of epoxy networks. ACS Sustain. Chem. Eng..

[56] Livi S., Silva A.A., Thimont Y., Nguyen T.K.L., Soares B.G., Gerard J.F., Duchet-Rumeau J. (2014). Nanostructured thermosets from ionic liquid building block-epoxy prepolymer mixtures. RSC Adv..

[57] Nguyen T.K.L., Livi S., Pruvost S., Soares B.G., Duchet-Rumeau J. (2014). Ionic liquids as reactive additives for the preparation and modification of epoxy networks. J. Polym. Sci. A Polym. Chem..

[58] Silva A.A., Livi S., Netto D.B., Soares B.G., Duchet J., Gerard J.F. (2013). New epoxy systems based on ionic liquid. Polymer.

[59] Binks F.C., Cavalli G., Henningsen M., Howlin B.J., Hamerton I. (2018). Investigating the mechanism through which ionic liquids initiate the polymerisation of epoxy resins. Polymer.

[60] Nguyen T.K.L., Livi S., Soares B.G., Benes H., Geerard J.F., Duchet-Rumeau J. (2017). Toughening of epoxy/ionic liquid networks with thermoplastics pased on Poly(2,6-dimethy1-1,4-phenylene ether) (PPE). ACS Sustain. Chem. Eng..

[61] Halawani N., Donato R., Benes H., Brus J., Kobera L., Pruvost S., Duchet-Rumeau J., Gerard J.-F., Livi S. (2021). Thermoset-thermoplastic-ionic liquid ternary hybrids as novel functional polymer materials. Polymer.

[62] Orduna L., Razquin I., Aranburu N., Guerrica-Echevarría G. (2022). Are ionic liquids effective curing agents for preparing epoxy adhesives?. Int. J. Adhes. Adhes..

[63] Heux L., Halary J.L., Laupretre F., Monnerie L. (1997). Dynamic mechanical and C-13 nmr investigations of molecular motions involved in the beta relaxation of epoxy networks based on DGEBA and aliphatic amines. Polymer.

[64] Smirnov Y.N., Glavina T.A., Efremova A.I. (2011). Effect of the molecular mobility of epoxy amine crosslinked polymers on their relaxation and physicomechanical characteristics. Polym. Sci. Ser. A.

[65] Lutzen H., Gesing T.M., Kim B.K., Hartwig A. (2012). Novel cationically polymerized epoxy/poly(ε-caprolactone) polymers showing a shape memory effect. Polymer.

[66] Iregui A., Irusta L., Martin L., Gonzalez A. (2019). Analysis of the process parameters for obtaining a stable electrospun process in different composition epoxy/Poly ε-caprolactone blends with shape memory properties. Polymers.

[67] Arnebold A., Wellmann S., Hartwig A. (2016). Network dynamics in cationically polymerized, crosslinked epoxy resins and its influence on crystallinity and toughness. Polymer.

[68] Remiro P.M., Cortazar M.M., Calahorra M.E., Calafel M.M. (2001). Miscibility and crystallization of an amine-cured epoxy resin modified with crystalline poly(ε-caprolactone). Macromol. Chem. Phys..

[69] Chen J.-L., Chang F.-C. (2003). Phase separation and melting behavior in poly(ε-caprolactone)-epoxy blends cured by 3,3′-dimethylmethylene-di(cyclohexylamine). J. Appl. Polym. Sci..

[70] Ratna D. (2003). Modification of epoxy resins for improvement of adhesion: A critical review. J. Adhes. Sci. Technol..

[71] Ratna D., Banthia A.K. (2000). Epoxidized soybean oil toughened epoxy adhesive. J. Adhes. Sci. Technol..

[72] Quan D., Murphy N., Ivankovic A. (2017). Fracture behaviour of a rubber nano-modified structural epoxy adhesive: Bond gap effects and fracture damage zone. Int. J. Adhes. Adhes..

[73] Quan D., Murphy N., Ivankovic A. (2017). Fracture behaviour of epoxy adhesive joints modified with core-shell rubber nanoparticles. Eng. Fract. Mech..

[74] Jin F.L., Park S.J. (2008). Impact-strength improvement of epoxy resins reinforced with a biodegradable polymer. Mater. Sci. Eng..

[75] Zhou H.Z., Liu H.Y., Zhou H.M., Zhang Y., Gao X.P., Mai Y.W. (2016). On adhesive properties of nano-silica/epoxy bonded single-lap joints. Mater. Des..

[76] Quan D., Carolan D., Rouge C., Murphy N., Ivankovic A. (2018). Mechanical and fracture properties of epoxy adhesives modified with graphene nanoplatelets and rubber particles. Int. J. Adhes. Adhes..

[77] Quan D., Carolan D., Rouge C., Murphy N., Lyankoyic A. (2017). Carbon nanotubes and core-shell rubber nanoparticles modified structural epoxy adhesives. J. Mater. Sci..

[78] Bascom W.D., Cottington R.L. (1976). Effect of temperature on adhesive fracture behaviour of an elastomer epoxy resin. J. Adhes..

[79] Achary P.S., Latha P.B., Ramaswamy R. (1990). Room temperature curing of CTBN-toughened epoxy adhesive with elevated-temperature service capability. J. Appl. Polym. Sci..

